# A model building exercise of mortality risk for Taiwanese women with breast cancer

**DOI:** 10.1186/1472-6947-10-43

**Published:** 2010-08-19

**Authors:** Tsai W Chang, Yao L Kuo

**Affiliations:** 1 Department of Surgery, National Cheng Kung University, College of Medicine, Tainan and Dou-Liou branch, Taiwan

## Abstract

**Background:**

The accurate estimation of outcome in patients with malignant disease is an essential component of the optimal treatment, decision-making and patient counseling processes. The prognosis and disease outcome of breast cancer patients can differ according to geographic and ethnic factors. To our knowledge, to date these factors have never been validated in a homogenous loco-regional patient population, with the aim of achieving accurate predictions of outcome for individual patients. To clarify this topic, we created a new comprehensive prognostic and predictive model for Taiwanese breast cancer patients based on a range of patient-related and various clinical and pathological-related variables.

**Methods:**

Demographic, clinical, and pathological data were analyzed from 1 137 patients with breast cancer who underwent surgical intervention. A survival prediction model was used to allow analysis of the optimal combination of variables.

**Results:**

The area under the receiver operating characteristic (ROC) curve, as applied to an independent validation data set, was used as the measure of accuracy. Results were compared by comparing the area under the ROC curve.

**Conclusions:**

our model building exercise of mortality risk was able to predict disease outcome for individual patients with breast cancer. This model could represent a highly accurate prognostic tool for Taiwanese breast cancer patients.

## Background

Breast cancer is a serious threat to women's health. In Taiwan, breast cancer ranked fourth among the top 10 causes of death among women in the period from 1995 to 2003 [1]. The investigative results published by the Bureau of Health Promotion, Department of Health, Taiwan, indicate that the incidence and mortality of breast cancer increase almost every year. The incidence rate and the age-adjusted incidence rate have both increased almost two-fold when compared with those calculated for the period from 1995 to 2003. The corresponding mortality also increased: the mortality rate increased from 8.9 per 10000 people to 12.45 per 10000 people and the age-adjusted death rate increased from 8.79 per 10000 people to 11.07 per 10000 people [2]. Improved surgical procedures and chemotherapy regimens seem not to have effectively diminished breast cancer incidence and mortality [3,4]. It is therefore important to identify risk factors that significantly affect survival among women with breast cancer, as the control of these risk factors.

Unlike most countries in Asia, which have produced few publications on cancer recurrence risk analyses among breast cancer patients, many such studies have been published in Western countries [5-8]. Among them, meta-analyses are widely used to discuss causal relationships between risk factors and breast cancer survival [9,10]. Meta-analyses are secondary analyses that derive results from data reported in different studies addressing similar research topics. A different combination of methods can lead to different meta-analytical outcomes. Furthermore, it is extremely difficult to predict the disease outcome of cancer patients. To solve this problem, we used a logistic regression approach to simultaneously investigate the relationships between all significantly effected risk factors, including demographic, clinical, and pathological data, and the survival status of breast cancer patients.

## Methods

The original data was collected from 1 190 patients with breast cancer diagnosed between January 1, 1995 and August 31, 2005 at the National Cheng Kung University Hospital, Tainan, Taiwan. As our objective was to study the prognostic factors of breast cancer and to develop more precise predictive mortality risk models, both patients with stage IV disease and patients who were followed up for less than one year were excluded from our analyses. Among the remaining 1 137 patients, 70 died and the other 1 067 were censored. The median age of the patients was 49 years (range, 20-88 years). Ethical approval was provided by Human Experiment and Ethics committee of the National Cheng Kung University Hospital (ER-99-076).

A variety of potential breast cancer risk factors were constructed for each patient. The demographic data included marriage status, education level, familial history of breast cancer, presence of other underlying diseases, and menopause status. The clinical data included physical examination (PE), ultrasound (US), fine-needle aspiration cytology (FNAC), core needle biopsy (CNB), mammography, type of breast surgery, and type of axillary lymphatic surgery. Finally, the pathological findings included tumor size, nodal status, tumor grade, estrogen receptor (ER) status, progesterone receptor (PR) status, Her-2/neu status, extensive intraductal carcinoma (EIC), presence of lymphatic tumor emboli (LTE), hepatitis B and C status, and hepatitis B surface antigen (HBsAg) and hepatitis C virus antibody (HCV Ab). The clinical and pathological data were classified into four categories: benign (B), intermediate (I), suspicious (S), and malignant (M). The different treatment modalities, including anti hormone therapy, radiotherapy, and chemotherapy, were also included in our analysis.

### Statistical methods

The overall survival function for breast cancer patients was calculated using the Kaplan-Meier method: the log-rank test was used to test the significance of different stage groups [11]. To investigate the association between survival status and each potential risk factor, odds ratios were computed and p values were evaluated by using univariate logistic regression test, where applicable [12]. Odds ratios were used to evaluate the relative odds of death caused by breast cancer between two groups sorted under a risk factor, and p values were calculated to assess significance of results. A multivariable logistic regression analysis was used to measure the significance of several risk factors simultaneously and to predict the survival probability of breast cancer patients [12]. To determine the accuracy of our model, Bootstrap method was used, which can be implemented by obtaining a number of re-samples of our observed dataset [13]. The predictive model, which was built using forward stepwise analyses, included only the risk factors that showed significance in the univariate analyses. Statistical significance was set at p < 0.05.

Three methods were used for the evaluation of the fitness of the multivariable logistic regression model. First, ROC curves (using FORTRAN programs) [14] were plotted to estimate the sensitivity and specificity of the predictive model. The closer that the area under ROC curve is to 1, the better the fit of the model. Second, the Hosmer-Lemeshow test, written as $\overset{\wedge }{C}=\sum_{k=1}^{g}\frac{{n^{\prime }}_{k}{\left(p_{k}-{\overset{\wedge }{p}}_{k}\right)}^{2}}{{\overset{\wedge }{p}}_{k}(1-{\overset{\wedge }{p}}_{k})}$ for the statistic being tested (where ${n^{\prime }}_{k}$ is the number of patients in the k^th ^group, and ${\overset{\wedge }{p}}_{k}$ and *p_k _*are the predicted and real possibilities of death, respectively, in the k^th ^group) was used to examine the fitness of the predictive model by considering the difference between the predicted and observed probabilities of death caused by breast cancer. Patients were divided into several groups according to ordered predicted probability of death. The statistic *Ĉ *is well approximated by the chi-square distribution with g-2 degrees of freedom, *X^2^_g-2_*. The larger the p value obtained using the Hosmer-Lemeshow test, (which corresponds to a smaller *Ĉ*), the smaller the square of the distance between ${\overset{\wedge }{p}}_{k}$ and *p_k_*, and hence, the better the fit of the model [15,16]. The comparison was performed based on the confidence interval of both models using the SPSS software, version 11.

## Results

**Figure 1:**
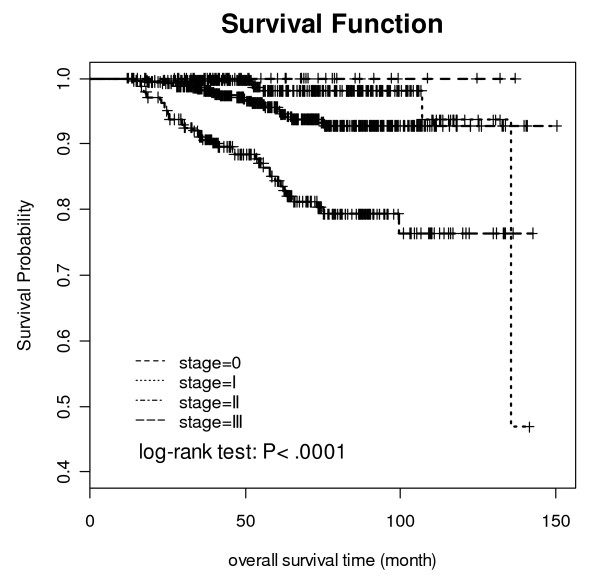
**The Kaplan-Meier survival curve in each stage of the patients**.

The overall median duration of patient follow-up was 60.3 months (range, 11.93-150.3 months). According to the staging rules of practice guidelines in oncology from the National Comprehensive Cancer Network, 70 patients (6.2%) patients had stage 0 disease, 310 patients (27.2%) had stage I disease, 506 patients (44.5%) had stage II disease, and 251 patients (22.1%) had stage III disease. The median duration of patient follow-up was similar for each stage (close to five years), with the exception of stage 0. The five-year survival probability for breast cancer was greater than 90%, and even for patients with stage III disease, the survival probability for five years was 84.33%. Log-rank testing showed that the differences in survival at the different stages were significant (p < 0.0001) (Figure 1).

### Associations of breast cancer mortality with demographic, clinical, and pathological factors

Patients for whom risk factor values were missing were excluded from the analytical process. Among the demographic data, only age and menopause correlated significantly with breast cancer survival. The mortality was lowest for patients between the ages of 36 and 57 years (4.5%). Conversely, patients aged from 20 to 35 years had the highest mortality (12%). The mortality difference for these two age groups was significant (p = 0.002). Regarding the menopausal status, the mortality of postmenopausal patients was higher than that of premenopausal patients, or of patients who had hysterectomy or oophorectomy (p = 0.0082); however, the effect of the menopause status on breast cancer mortality could be a reflection of the age of the patients. (Table 1) The analysis of the clinical data revealed that all clinical risk factors were correlated with survival status. (Table 2) In what concerns the pathology data, the survival rate did not correlate with hepatitis status, HBsAg, or HCV Ab. In contrast, the following pathology outcomes were positively associated with increased breast cancer mortality: higher tumor grade (p < 0.0001), negative ER status (p = 0.0086), negative PR status (p = 0.0086), positive Her-2/Neu status (p = 0.0137), absence of EIC (p = 0.0323), presence of LTE (p = 0.0004), increased tumor size (p < 0.0001), axillary lymph nodes (p < 0.0001), and abandonment or refusal of chemotherapy treatment (p < 0.0001) (Table 3).

**Table 1:** Description of the population by univariate logistic regression test using the demographic data

	**Demographic Data**		
Factor	Death(%)	Odds Ratio	P
**Marital status**		1.334	N.S.
Married (n = 1071)	67 (6.3%)		
Unmarried (n = 63)	3 (4.8%)		
**Education level**		1.677	0.057
Below junior high school (n = 685)	50 (7.3%)		
Above senior high school (n = 446)	20 (4.5%)		
**Menopause**		1.925	0.008 *
Premenopause (n = 397)	35 (8.8%)		
Others (n = 732)	35 (4.8%)		
**Hormone use**		1.319	N.S.
No (n = 798)	40 (5.0%)		
Yes (n = 104)	4 (3.8%)		
**Familial breast cancer history**		0.732	N.S.
No (n = 997)	59 (5.9%)		
Yes (n = 139)	11 (7.9%)		
**Underlying diseases**		0.713	N.S.
No (n = 577)	30 (5.2%)		
Yes (n = 560)	40 (7.1%)		
**Age (years)**		0.378 (a v.s. c)	0.002 *
		0.745 (b v.s. c)	N.S.
36-60 (a) (n = 856)	42 (4.9%)		
61-85 (b) (n = 206)	19 (9.2%)		
20-35 (c) (n = 75)	9 (12%)		

Menopause: others, excluding premenopase, hysterectomy, and s/p ovarian.

Age: others, concluding patients more than 58 or less than 36.

N. S.: Statistically not significant

**Table 2:** Description of the population by univariate logistic regression test using the clinical data

	**Clinical Data**		
Factor	Mortality(%)	Odds Ratio	P-value
**Physical examination**		0.304 (a v.s. c)	0.002 *
		0.616 (b v.s. c)	N.S.
N, B, I (a) (n = 484)	16 (3.3%)		
S (b) (n = 216)	14 (6.5%)		
M (c) (n = 267)	27 (10.1%)		
**Ultrasound**		0.279	<.0001 *
N, B, I, S (n = 741)	27 (3.6%)		
M (n = 251)	30 (12%)		
**FNAC**		0.375	0.009*
B, I, S (n = 323)	9 (2.8%)		
M (n = 591)	42 (7.1%)		
**Mammography**		0.269	0.0001 *
BR1-4 (n = 446)	11 (2.5%)		
BR5 (n = 512)	44 (8.6%)		
**Diagnostic** **method**		0.429	0.0008 *
Core biopsy (n = 661)	27 (4.1%)		
Others (n = 476)	43 (9.0%)		
**Breast surgery**		0.063	0.0001 *
BCS (n = 341)	2 (0.6%)		
TM (n = 795)	68 (8.6%)		
**Lymphatic surgery**		0.237	0.0004 *
SLNB (n = 341)	7 (2.1%)		
ALND (n = 775)	63 (8.1%)		

Diagnostic: others, concluding combined methods, Excision biopsy, and FS

N. S.: Statistically not significant

**Table 3:** Description of the population by univariate logistic regression test using the pathological data

	**Pathological Data**		
Risk factors	Mortality(%)	Odds Ratio	P-value
**Pathological report**		<.001 (a v.s. c)	N.S.
		0.532 (b v.s. c)	N.S.
Others (a) (n = 138)	0		
Invasive ductal carcinoma (b) (n = 966)	66 (6.8%)		
Invasive lobular carcinoma (c) (n = 33)	4 (12.1%)		
**Tumor grade**		2.172	<.0001 *
III (n = 234)	27 (11.5%)		
II (n = 431)	24 (5.6%)		
I (n = 294)	8 (2.7%)		
**ER/PR**		1.99	0.009 *
(-, -), (-, +), (+, -) (n = 583)	47 (8.1%)		
(+, +) (n = 545)	23 (4.2%)		
**Her-2/Neu**		2.125	0.014*
+++ (n = 181)	18 (9.9%)		
-, +, ++ (n = 668)	33 (4.9%)		
**EIC**		2.036	0.032 *
Absent (n = 590)	46 (7.8%)		
Present (n = 301)	12 (4.0%)		
**LTE**		2.856	0.0004 *
Present (n = 501)	46 (9.2%)		
Absent (n = 468)	16 (3.4%)		
**HBsAg**		1.22	N.S.
+ (n = 124)	8 (6.5%)		
- (n = 598)	32 (5.4%)		
**HCVAb**		1.103	N.S.
+ (n = 50)	3 (6%)		
- (n = 658)	36 (5.5%)		
**R/T**		1.002	N.S.
Yes (n = 581)	39 (6.7%)		
No (n = 552)	31 (5.6%)		
**Anti-hormone therapy**		1.002	N.S.
No (n = 228)	14 (6.2%)		
Yes (n = 909)	56 (6.2%)		
**Tumor size**		2.579	<.0001 *
T4 (n = 57)	10 (17.5%)		
T2 or T3 (n = 595)	45 (7.6%)		
Tis or T1 (n = 485)	15 (3.1%)		
**Node**		4.053	<.0001 *
N3 (n = 81)	23 (28.4%)		
N1 or N2 (n = 377)	31 (8.2%)		
N0 (n = 677)	16 (2.4%)		
**Chemotherapy**		0.061 (a v.s. c)	<0.0001*
		0.391 (b v.s. c)	0.009 *
No (a) (n = 327)	4 (1.2%)		
Yes (b) (n = 745)	55 (7.4%)		
Abandonment or Refusal (c) (n = 65)	11 (18%)		

N. S.: Statistically not significant

### Multivariable logistic regression

Of the original 1 067 patients, 818 patients with complete data were included in the multivariable logistic regression analysis. Among them, 43 patients died and 775 were censored. As shown in Table 4, the odds ratio for patients aged 36-60 years versus patients aged 20-35 years is 0.254, which means that the odds of death for a patient in the latter age group is approximately four times (1/0.254) greater than that for patients in the former age group (p = 0.0029). The odds ratio of patients with an ultrasound examination showing malignancy were also around two times higher than those with benign, intermediate, or suspicious ultrasound results (odds ratio = 2.028, p < 0.0001). In what concerns the remaining four chosen pathological risk factors, the odds of death were positively correlated with a higher tumor grade (odds ratio = 1.626, p = 0.01) or lymph node involvement (odds ratio = 3.054, p < 0.0001): the odds increased about two times when the tumor grade was II versus grade I, or grade II versus grade III, and three times when lymph node status was N1 or N2 versus N0 or N3 versus N1 or N2. Patients who abandoned or refused chemotherapy had approximately three times greater odds of death than patients who completed chemotherapy treatment (odds ratio = 0.242, p = 0.0016). Compared to the univariate logistic analysis, multivariable logistic analysis and Bootstrap for variables showed only difference in the patient group examined with ultrasound (p = < 0.0001, <0.0001 and 0.111 respectively).

**Table 4:** Comparison of risk factors calculated using the univariate logistic analysis, multivariable logistic analysis and Bootstrap for variables.

	**Univariate logistic analysis**			**Multivariate logistic analysis**			**Bootstrap for Variables in the Equation**		
**Factors**	**Odds Ratio**	**95% CI**	**P-value**	**Odds Ratio**	**95% CI**	**P-value**	**Std. Error**	**95% CI**	**P-value**

**Age (years)**									

36-60 (a)	2.646 (c v.s. a)	(1.232, 5.650)	**0.002**	3.937 (c v.s. a)	(1.403, 11.111)	**0.003**	0.892	(0.945, 12.195)	**0.013**

61-85 (b)	1.342 (c.v.s. b)	(0.579, 3.115)		1.548 (c v.s. b)	(0.492, 4.878)		0.924	(0.370, 5.348)	

20-35 (c)									

**Ultrasound**									

M	3.584	(2.088, 6.173)	**<0.0001**	1.977	(1.545, 4.367)	**<0.0001**	0.471	(1.023, 4.993)	**0.111**

N, B, I, S									

**Mammography**									

BR5	3.717	(1.898, 7.299)	**0.0001**	3.058	(1.049, 8.929)	**0.048**	1.315	(1.121, 1.530)	**0.028**

BR1-4									

**Diagnostic method**									

Others	2.331	(1.422, 3.846)	**0.001**	2.519	(1.172, 5.410)	**0.028**	0.427	(1.119, 6.303)	**0.016**

Core biopsy									

**Tumor grade**									

III	2.172	(1.486, 3.176)	**<0.0001**	1.671	(1.215, 2.829)	**0.01**	0.322	(0.966, 3.347)	**0.075**

II									

I									

**Lymph node**									

N3	4.053	(2.850, 5.755)	**<0.0001**	3.037	(1.808, 5.104)	**<0.0001**	0.335	(1.624, 6.221)	**0.001**

N1 or N2									

N0									

**ER/PR**									

(-, -), (-, +), (+, -)	1.99	(1.194, 3.331)	**0.009**	2.778	(1.231, 6.260)	**0.045**	0.451	(1.306, 7.996)	**0.011**

(+, +)									

**Chemotherapy**									

Yes (a)	2.558 (c v.s. a)	(1.263, 5.155)	**<0.0001**	4.348	(1.631, 11.494)	**0.002**	0.852	(1.339, 13.514)	**0.003**

No (b)	16.393 (c v.s. b)	(5.051, 52.632)		12.658	(2.558, 62.5)		3.761	(18.868, 2.87*10^9^)	

Abandonment or Refusal (c)									

### Goodness of fit

**Figure 2:**
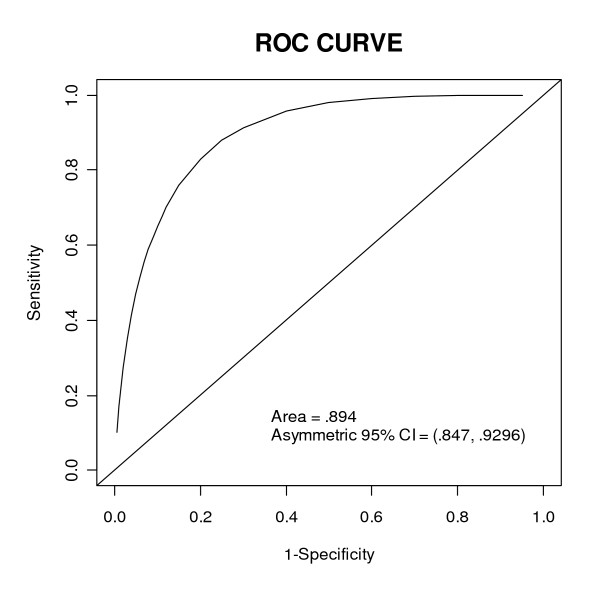
**ROC curve calculated by the multiple logistic regression model**.

**Figure 3:**
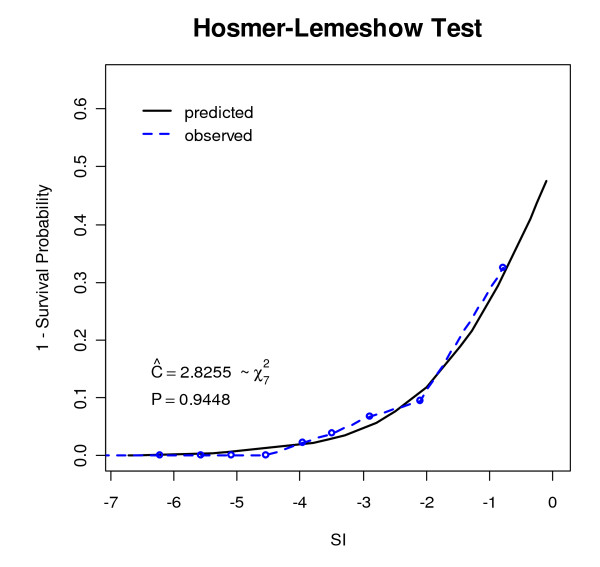
**The curve of the predicted and observed probability of death**.

Our model showed a good fit based on both the ROC curve and the Hosmer-Lemeshow test. The area under the ROC curve was 0.894 with asymmetric confidence interval equals (0.8405, 0.9318), not concluding 0.5. The larger area, farther from 0.5 and closer to 1, showed the excellence of the model's performance. Best cutoff value showed p = 0.0419, sensitivity = 0.86, specificity = 0.756, positive predictive value (PPV) = 0.1889 and negative predictive value (NPV) = 0.9879 (Figure 2). The Hosmer-Lemeshow test also showed excellent performance of the model (p = 0.9448) (Figure 3). The curve of the observed probability of death is much closer to that of the predicted probability of death.

## Discussion

In recent years, several improvements in medical treatment modalities in breast cancer were observed; [4,17-19] however, the overall prognosis and predictive values for breast cancer patients remains ambiguous [5,6]. It is important to improve the efficiency of predicting the survival of breast cancer patients; therefore, a model building exercise can be extended to include any number of prognostic or risk factors, while also providing treatment predictions. The TNM classification system has long been the accepted predictive tool for breast cancer and provides useful information for the clinical decision-making process in these patients; [20] however, this system is based solely on disease-related parameters and does not include diverse variables, including diagnostic methods, which may influence the outcome of patients. Furthermore, a comprehensive predictive model should also take into account treatment modalities, including chemotherapy, hormone therapy or targeted therapy, which are currently either in use or are under study [21-23].

The impact of the race of the individual on the survival of breast cancer patients has been reported [24-26]. To our knowledge, the current study is the first to demonstrate systematically the influence of prognostic factors on the survival of patients with breast cancer in Asian and Pacific Islander populations. Admittedly, the design of the model and the selection of variables should have considerable clinical applicability. The main purpose of our study was to construct a suitable survival prediction formula for Taiwanese women. To create a survival prediction model for breast cancer, we used a comprehensive dataset that included clinico-pathological data, diagnostic modalities, and treatment variables from Taiwanese patients who suffering from this disease.

Our model building exercise showed that age, ultrasound diagnostic classification, mammography diagnostic classification, diagnosis by core biopsy, tumor grade, ER/PR status, lymph node status, and chemotherapy are the most important predictive factors for breast cancer in Taiwanese women. The combination of these risk factors using multivariable logistic regression analysis, led to the development of a predictive formula for breast cancer survival. Our data also draw attention to the importance and influence of diagnostic modalities on breast cancer survival rate. In our model building exercise, the use of ultrasound, mammography, and core biopsy technologies had a high impact on disease outcome.

The prognostic power in a disease context can be improved by applying a predictive model, even when using TNM data or other predictive factors [7,22,27]. Burke et al. [28] demonstrated that the predictive accuracy for breast and colon carcinoma could be improved by using an ANN-based model using TNM information exclusively. Similarly, in the current study we created an additional model for the prediction of survival in patients with breast cancer using data that was more complete than TNM staging information.

The high predictive accuracy of the current model may stem from several factors. First, in other models, investigators often relied strongly on input data that were weighted toward tumor histopathological parameters, rather than toward clinical or demographic patient data [6,17,19,21,27]. This is in contrast to the current model, in which several parameters, including diagnostic and treatment modalities or demographic data, represented the majority of the selected optimal variable datasets. Second, the current study is the first to use prognostic factors as a predictive tool in Asian breast cancer populations.

Caution should nevertheless be employed when generating and interpreting data using our model building exercise. First, the current model was based on data assembled at a single institution; therefore, the validity of this model should be verified before its application to patients from other populations or institutions. The variability in survival rates observed for breast cancer patients from different countries seems to support this argument [25,26]. A possible method for overcoming this limitation may be the inclusion of patients from other Asian populations in the construction of a new model. Thus, the identification and evaluation of universally applicable variables may require collaborations between different institutions or nations. Nevertheless, the current pilot study serves as a proof-of-principle strategy that underscores the utility of this model building exercise. Second, the data used here were not established from prospective and randomized studies. If other users wish to adopt our model building exercise for the selection of therapeutic methods, then any variables pertaining to focused treatment methods should be compared with standardized protocols. If treatment variables were included, any result would be biased by case-by-case selection criteria for that particular treatment; [7,8] therefore, a web-based prediction engine may facilitate its use by clinicians in the future.

## Conclusions

We have designed an effective model for predicting outcomes in Taiwanese breast cancer patients by combining demographic, clinical, and pathological data, including multiple tumor-related and patient-related variables. Our model building exercise showed a strong potential to enhance the prediction of patient survival and to identify important variables that have an impact on disease outcomes. Information provided by this model building exercise may improve the selection of appropriate and effective therapy for breast cancer patients.

## Competing interests

The authors declare that they have no competing interests.

## Authors' contributions

CTW designed the concept of this study, drafted the manuscript and performed treatment. CTW collected the data and performed the statistical analysis. KYL approved the final manuscript. KYL designed the concept of this study and provided treatment coordination. All authors read and approved the final manuscript.

## Pre-publication history

The pre-publication history for this paper can be accessed here:

http://www.biomedcentral.com/1472-6947/10/43/prepub
